# Sequence based model using deep neural network and hybrid features for identification of 5-hydroxymethylcytosine modification

**DOI:** 10.1038/s41598-024-59777-y

**Published:** 2024-04-20

**Authors:** Salman Khan, Islam Uddin, Mukhtaj Khan, Nadeem Iqbal, Huda M. Alshanbari, Bakhtiyar Ahmad, Dost Muhammad Khan

**Affiliations:** 1 Department of Computer Science, Abdul Wali Khan University Mardan, Mardan, Pakistan; 2 Department of Information Technology, The University of Haripur, Haripur, Pakistan; 3 Department of Mathematical Sciences, College of Science, Princess Nourah bint Abdulrahman University, P.O. Box 84428, 11671 Riyadh, Saudi Arabia; 4 Higher Education Department Afghanistan, Kabul, Afghanistan; 5 Department of Statistics, Abdul Wali Khan University Mardan, Mardan, 23200 KP Pakistan

**Keywords:** 5-Hydroxymethylcytosine, RNA, Cancer, Diabetes, Cardiovascular, Deep learning, Engineering, Computer science, Scientific data, Statistics

## Abstract

RNA modifications are pivotal in the development of newly synthesized structures, showcasing a vast array of alterations across various RNA classes. Among these, 5-hydroxymethylcytosine (5HMC) stands out, playing a crucial role in gene regulation and epigenetic changes, yet its detection through conventional methods proves cumbersome and costly. To address this, we propose Deep5HMC, a robust learning model leveraging machine learning algorithms and discriminative feature extraction techniques for accurate 5HMC sample identification. Our approach integrates seven feature extraction methods and various machine learning algorithms, including Random Forest, Naive Bayes, Decision Tree, and Support Vector Machine. Through K-fold cross-validation, our model achieved a notable 84.07% accuracy rate, surpassing previous models by 7.59%, signifying its potential in early cancer and cardiovascular disease diagnosis. This study underscores the promise of Deep5HMC in offering insights for improved medical assessment and treatment protocols, marking a significant advancement in RNA modification analysis.

## Introduction

A single-stranded molecule called RNA is vital in producing cellular proteins and acts as a carrier of genetic information. RNA molecules exist in all living things, and they are fundamentally composed of a complex collection of molecules that carries genetic information and instructions that are essential for the development and maintenance of organisms^1^. The RNA molecules also move genetic information and instruction through certain viruses^2^. Several studies reported that more than 100 types of RNA alterations have been identified that have changed the structure and functions of RNA molecules^2–4^. For example, N6-Methyladenosine modifications and N7-Methylguanosine have been found in mRNA, and both control every phase of the lifecycle of the mRNA molecule^5^. Similarly, 5-methylcytosine and N1-methyladenine RNA modifications have been found in transfer RNA(tRNA) and ribosomal RNA (rRNA) molecules^6,7^. Another type of modification is 5HMC, which is produced through TET oxidation^8^.

The initial identification of the 5HMC alteration came from the study of wheat seeds, revealing a profound discovery that transcends the limitations of life, manifesting across a wide range of species and diverse domains with remarkable breadth and depth. The 5HMC alteration's pervasive influence has shed light on its pervasive influence, permeating the fabric of biological existence and heralding a new era of understanding in the intricate tapestry of genetic phenomena^9^. Furthermore, the 5HMC modification is also present in various tissues of both humans and mice, exerting a significant role in numerous genetic processes, including RNA splicing, RNA translation, and RNA decay. The 5HMC modification's influence extends across different biological contexts, underscoring its relevance in the intricate mechanisms governing genetic regulation and expression^10^. Similarly, the complex environment of 5hmC modifications intertwines with various human ailments, forging consequential links to multiple disorders that plague our species, including the formidable realms of cancer, diabetes, and cardiovascular disease. These findings highlight the significant vital role of 5hmC modifications in the complex web of human health, beckoning the pursuit of transformative insights for medical knowledge advancement and the development of targeted therapeutic interventions^11^. Detecting 5hmC involves a variety of methodological approaches in biochemistry and chemical properties, including chromatography (LC–MS/MS, HPLC, TLC)^12^. These techniques shed light on the enigmatic presence of 5hmC with precision. PCR amplification complements chromatography in illuminating the intricate tapestry of 5hmC. Together, these methods empower the scientific community to explore 5hmC, unravel its mysteries, and shape biological understanding^13^. Even though they yield effective results, they are highly costly and time-consuming for identifying 5hmC.

In recent years, there has been a significant increase in studies suggesting different techniques based on machine learning for identifying and describing the elusive 5HMC sites. For instance, Liu et al.^14^ presented a novel machine-learning approach that supports vector machine (SVM) algorithms, a newly developed sequence-based feature extraction technique, and other sophisticated methods. The classification of 5 hmC sites was revolutionized by this cutting-edge approach, which also gave previously unattainable insights into the intricate world of epigenetic modifications. Similarly, Ahmed et al.^12^ proposed developing the iRNA5hmC-PS. This model combined the most advanced Position-Specific Gapped k-mer (PSG k-mer) technique for feature extraction with the robust Logistic Regression algorithm for classification. Although the models outlined previously have shown promising results, it is important to remember that they rely on traditional learning processes. Because of their surprising resemblance, these models fail to predict 5hmC sequences correctly. Extracting the dominant features necessitates extensive human expertise and computational capabilities, adding to the task's complexity^12,14^. In a recent research study, Ali et al.^15^ proposed a revolutionary iRhmC5CNN model that employs Convolutional Neural Networks (CNNs) for efficient 5hmC identification. The authors used a one-hot-encoding technique within the CNN model to extract significant features. However, when applied to image datasets, this CNN-based model achieved outstanding performance, demonstrating its versatility and potential in the field.

**Figure 1:**
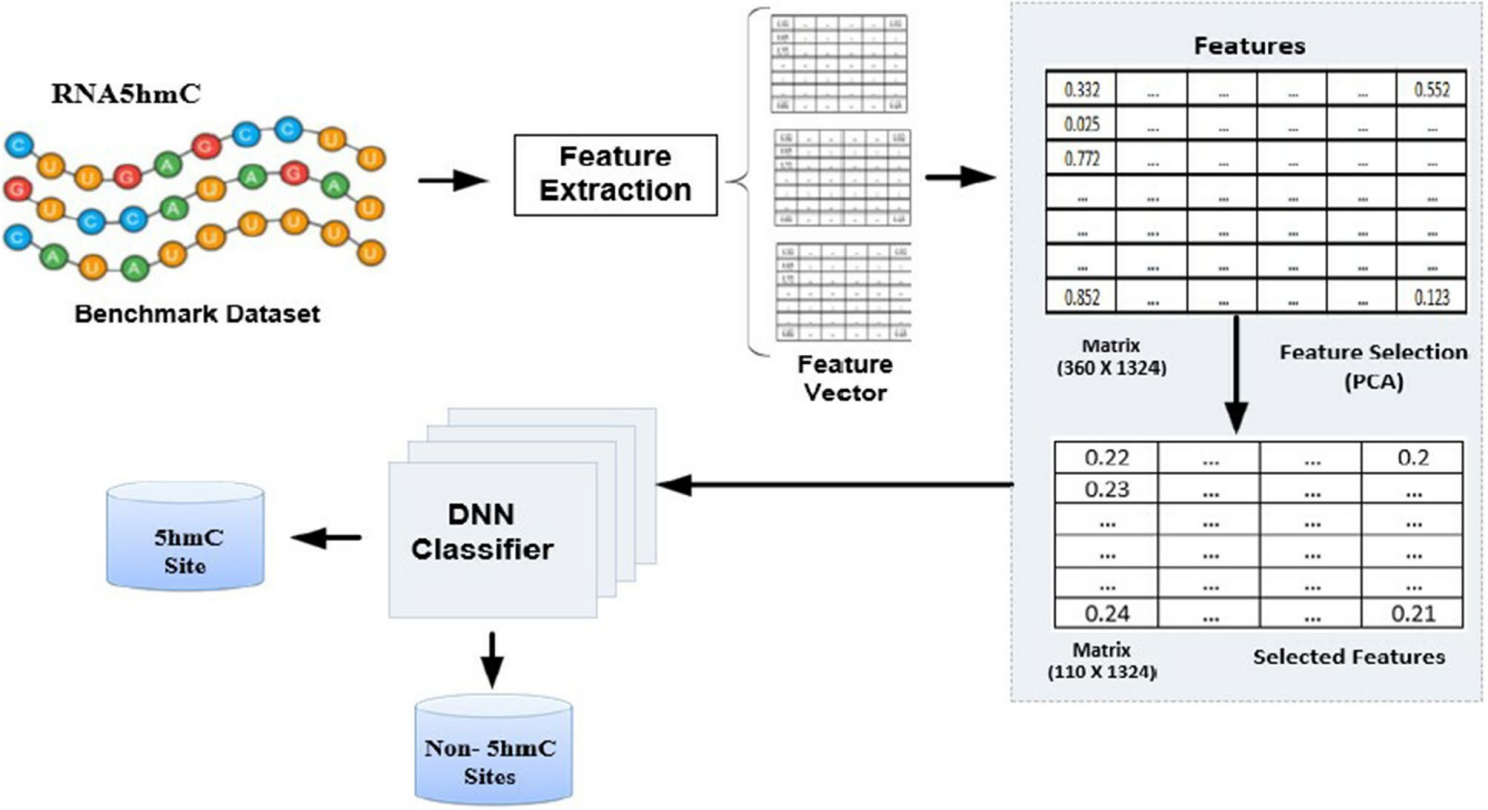
The architecture of the suggested predictor.

Based on the above previous studies, this study presents a novel and accurate model, utilizing Chou's comprehensive 5-step rules and regulations, discriminative feature methods, and the powerful Deep Neural Network (DNN) algorithm. The main objective of this research is to enhance the robustness and prediction accuracy of the model^16,17^. The intuition of the suggested model to improve the prediction accuracy of 5HMC modification. The workflow of the proposed model is depicted in Fig. 1. Firstly, the Deep5HMC employs seven different feature extraction methods to formulate RNA sequences into feature vectors. Secondly, a composite feature vector is constructed by combining all the feature vectors. Thirdly, it employs an unsupervised PCA method to select optimum features while removing irrelevant and repeated features. Finally, the computational model presented in this study employs a deep learning algorithm, suggesting a significant advancement in the field. Deep5HMC performance has been completely evaluated, with robust K-fold cross-validation tests used to establish the reliability of the study outcomes. The experimental results unambiguously demonstrate the proposed model's advantage in existing prediction models, as it demonstrates exceptional accuracy and consistently outperforms other performance measurement parameters by a significant margin, establishing its position as a leading solution in computational simulation. This study's benchmark datasets and feature vectors are easily accessible via the following GitHub link: https://github.com/salman-khan-mrd/Deep5HMC. This archive is a comprehensive resource for researchers and practitioners interested in diving into the complexities of Deep5HMC and its potential applications.

The remainder of this paper is organized in the following order: Section “Material and methods” describes the suggested model materials and methods in detail. Section “Feature extraction methodology” presents the paper various evaluation metrics. Section “Hybrid feature extraction” addresses the paper's experimental results, and Section “Criteria for performance evaluation” provides the paper conclusion and future work.

## Material and methods

### Benchmark dataset

It is imperative to have a comprehensive benchmark dataset containing training and test samples, each with a specific purpose to establish a highly effective learning model. The training samples are used to train the learning model, while the test samples are utilized to evaluate and validate the proposed model's performance. This study used a publicly available benchmark dataset^18^ with diverse samples. Equation (1) represents the mathematical formulation of this benchmark dataset, which includes both positive and negative examples, providing an excellent basis for our research

1 $${H}_{1} = {H}_{1}^{+}\cup {H}_{1}^{-}$$

where ${H}_{1}$ denoted the total number of RNA samples that contain positive samples ${H}_{1}^{+}$ and negative samples ${H}_{1}^{-}$. U is a mathematical operator representing the union of the 5-hmC positive and 5-hmCnegative samples. We also used CD-HIT software to remove pairwise sequences with greater than 20% similarity. Finally, the utilized benchmark dataset in this study comprises 1324 samples, divided into two categories: 662 positive samples that represent 5hmC sequences and 662 negative samples that represent non-5hmC sequences. Additionally, to ensure proper stratification, after randomly selecting 10% of the samples from our initial dataset, we built an independent dataset. The remaining 90% of the samples were employed to construct and evaluate the proposed model. The formulation of this independent benchmark dataset is mathematically captured by Eq. (2), thereby encapsulating the essence of our research findings and contributing significantly to a profound understanding of the proposed model.

2 $${H}_{2} = {H}_{2}^{+}\cup {H}_{2}^{-}$$

where, ${H}_{2}$ represents a test dataset that contains positive samples ${H}_{2}^{+}$ and negative samples ${H}_{2}^{-}$. The test dataset contains 132 samples in total, providing a substantial and representative collection of data points for comprehensive evaluation and analysis.

## Feature extraction methodology

Processing biological samples with varying lengths or non-numeric formats, such as the FASTA format, presents a challenge due to the specific nature of statistical and machine learning models designed to handle numerical data. Consequently, a crucial pre-processing step becomes necessary to convert RNA samples into feature vectors, representing discrete or numeric representations. This conversion ensures compatibility with learning models. It should be noted that the initial arrangement sequence or pattern of biological sequences might change during the transformation process. Several techniques for addressing this issue have been offered in prior studies. These approaches are intended to accelerate converting RNA samples into feature vectors while retaining the samples' organic patterns and structures. For this investigation, we successfully turned RNA samples into their corresponding feature extraction vectors using seven unique feature extraction methodologies.

### Descriptor K-mer

In the K-mer descriptor approach^19^, the average frequencies of the K-neighbouring nucleic acids are employed to encode the RNA samples. This method entails the data being encoded in a K-mer description, emphasizing the case where $k = 3.$ To provide a mathematical expression of this concept, we articulate the K-mer descriptor in the following:

3 $${R }_{Kmer}= f(t) * \frac{R(t)}{R}, t \in \{AAA, AAC, AAG, ..., AAU\}$$

where $R(t)$ represents the whole number of K-mer types, $t$ represents a particular K-mer sequence, and $R$ indicates the total number of sequences and the average number of the K-mer sequence.

### Reverse complement K-mer (RC-Kmer)

The RC-Kmer descriptor^20^ is a different approach to the K-mer description that dismisses the exact positioning of the strands. For example, there are 16 types of 2-mers $AA, AC, AG..., UU$ that act as a reverse complement to the letters $AA$ in an RNA sample. After removing these reverse complement k-mers, we are left with only ten different reverse complement k-mers $AC, CC, GC, AG, CG, and AU$. The reverse complement K-mer can be expressed mathematically using Eq. (4).

4 $${R}_{RcKmer}\left(r,s,t\right)\frac{{R}_{r,s,t}}{R-2}, r,s, t\in \left\{A, C, G,U\right\}$$

### Pseudo K-tuple nucleotide composition (PseKNC)

In computational biology, the popular feature extraction technique Pseudo K-Tuple Nucleotide Composition (PseKNC) allows RNA and DNA sample modification^21^. We used PseKNC with two different values of K to convert RNA samples into feature vectors: $PseDNC (K = 2)$ and $PseTNC (K = 3)$. PseDNC generates a 16-dimensional feature vector from RNA samples represented as pairs of subsequent nucleotides^21,22^. PseTNC, on the other hand, represents the RNA sequence with three consecutive nucleotides, resulting in a 64-dimensional feature vector. It is also mathematically expressed as follows:

5 $$R = [f_{1}^{K - tuple} f_{2}^{K - tuple} . \, ...... f_{i}^{K - tuple} .\, ....... f_{{4^{k} }}^{K - tuple} ]^{T}$$

where,

6 $${R}_{PseDNC}=|{f}_{j=1,...16D}^{2-Tuple} \quad \quad f \to (AA,CC,GG,UU)$$

7 $${R}_{PseTNC}=|{f}_{j=1,...64D}^{3-Tuple} \quad \quad f \to (AAA,CCC,GGG,UUU)$$

### Tri-nucleotide-based auto covariance (TAC)

A relevant encoding strategy is tri-nucleotide-based autocovariance (TAC), which is employed to examine the relationship between two tri-nucleotides. These tri-nucleotides are separated by a specified lag $L$ of nucleic acids along the sequence^23^. The total allowed capacity (TAC) is calculated using the following mathematical expression:

8 $${R}_{TAC}={\sum }_{j=1}^{L-2} \frac{{R}_{j}+ 2 }{L-2}$$

### Tri-nucleotide-based cross covariance (TCC)

Using a combination of tri-nucleotides separated by a predefined lag $L$ of nucleic acids throughout the sequence, the Tri-nucleotide-based Cross Covariance (TCC) encoding approach evaluates the connection between two different physicochemical parameters. TCC encoding may be calculated using the following equation:

9 $${R}_{TCC}={\sum }_{j=1}^{L-2}\frac{{R}_{j}+ 2 }{L-2}$$

### Dinucleotide-based cross covariance (DCC)

The relationship between two different physicochemical qualities encompassing a variety of di-nucleotides spaced by a specific nucleic acid lag $L$ within the sequence is investigated using DCC encoding^19^. Important details may be found in the text below that can be utilized to calculate DCC encoding:

10 $${R}_{DCC}={\sum }_{j=1}^{L-1}\frac{{R}_{j}{R}_{j}+1}{L-1}$$

The mathematical formula $R({R}_{1})$, LAG determines the feature vector connected to the DCC dimension. R is the number of physicochemical parameters, and LAG signifies the most important lag value (lag = 1, 2…, LAG). This equation, which considers numerous physicochemical characteristics and lag values, accurately estimates the feature vector's size. This equation may be used to precisely assess the dimensions of the feature vector, which include the numerous extended lag values and physicochemical indices studied.

## Hybrid feature extraction

In this study, we used a variety of seven feature extraction techniques to represent the 5hmC sequence precisely as a numerical feature vector. The summarized results in Table 1 provide specific details on the feature numbers achieved for each method. We effectively combined all seven feature vectors, leading to the creation of an expanding hybrid feature vector by using Eq. (11), as well as the smooth integration of Eqs. (3)–(4) and (6)–(10). This thorough rendering captures the core of the 5hmC sequence and makes it easier to analyze it in terms of numerical components.

**Table 1:** Number of features of each feature extraction method.

Methods	Number of features
Kmer	16
RC-Kmer	10
PseDNC	16
PseKNC	64
TAC	04
TCC	04
DCC	60

11 $${R}_{HFV}={R}_{Kmer}\cup {R}_{RcKmer}\cup {R}_{PseDNC}\cup {R}_{PseTNC}\cup {R}_{TAC}\cup {R}_{TCC}\cup {R}_{DCC}$$

### Feature selection using principal component analysis (PCA)

The feature vector's presence of noisy, duplicated, or irrelevant information may severely impact the classifier's performance. Therefore, we use the Principal Component Analysis (PCA) technique for feature selection. The PCA algorithm is a multivariate data processing technique that computes eigenvectors and covariance matrices to decrease the dimensionality of feature vectors. In this study, PCA is used to improve the precision of the selected feature vectors taken from RNA sequences used by Deep5HMC for model training. PCA reduces the dimensionality from the original high-dimensional feature space to a refined subspace of lower dimensions in which it still retains the most significant features but eliminates most of the redundant and noisy information. This process, in turn, allows for a more declarative representation of the data, which tends to avoid complexity and the risk of fitting too much. Significantly, in our methodology, feature vectors formed from the extraction method of 7 different hybridized techniques are sent through the PCA unsupervised selection procedure, and optimum features are chosen. Using the PCA technique, we successfully reduced the size of the hybrid feature vector from 174 × 1324 to 75 × 1324, resulting in a clearer and more effective representation. These things yield feature vectors that still have important data, and so the Deep5HMC model has high accuracy and interpretability in the 5HMC modification using RNA patterns as predictors.

### Deep neural network

**Figure 2:**
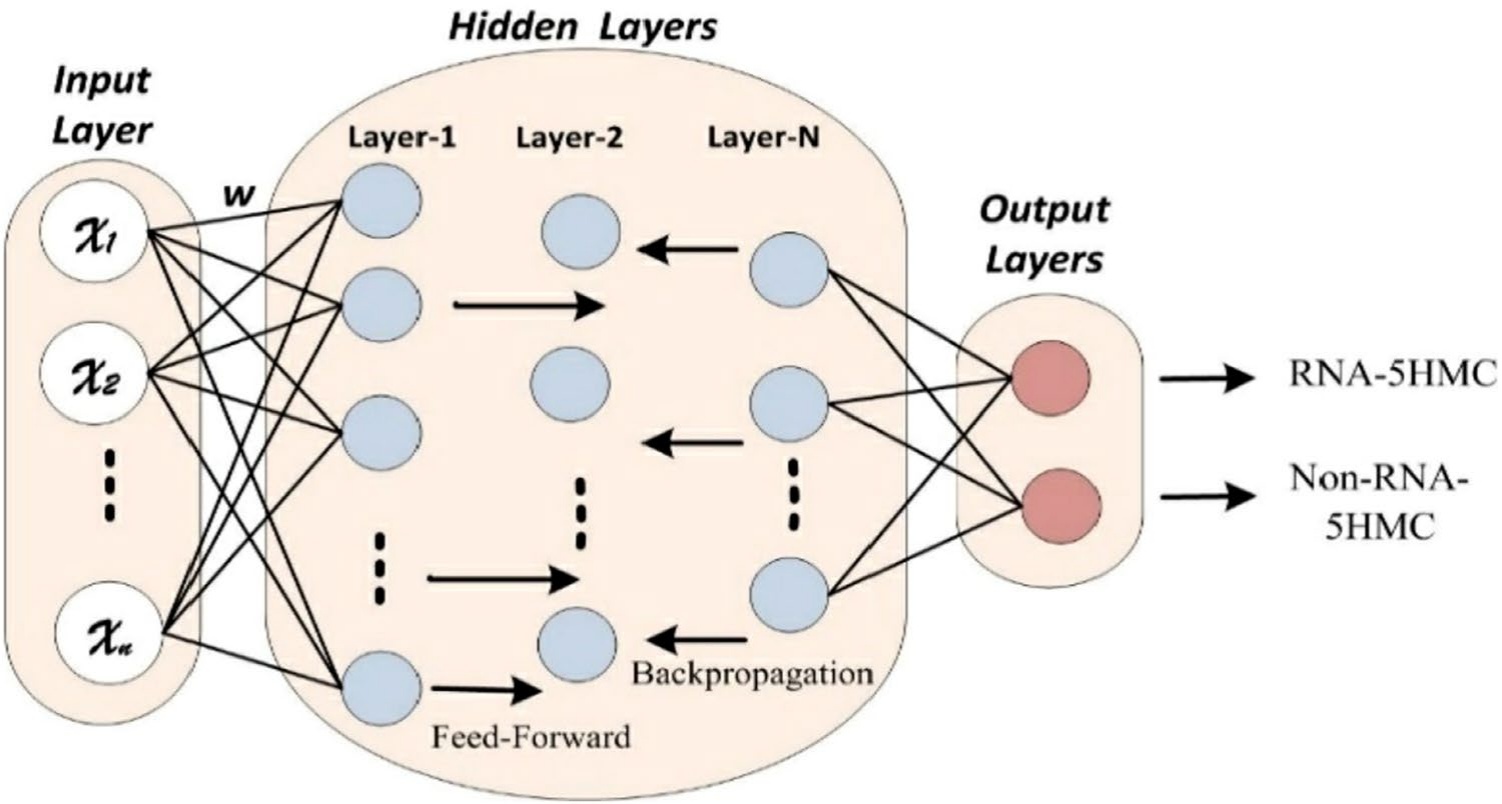
The configuration of the proposed DNN Mode circle represents processing nodes.

Deep Neural Network (DNN), a novel learning algorithm inspired by the intricate intricacy of the human neuron system, has emerged as an extremely powerful framework^24,25^. As shown in Fig. 2, this complete model includes three important processing levels: the input, hidden, and output layers. Hidden layers in the DNN model are important for the complicated learning process^26–28^. It is important to carefully consider the possible disadvantages of using a full-layer architecture, such as increased computational costs, increased complexity of the model, and the possibility of over-fitting, even though the number of hidden layers has an inherent impact on model performance^29^. Several studies have consistently shown that the DNN model performs better than classical learning techniques, especially when faced with various challenging classification problems^30^. Additionally, the DNN model has proven to be incredibly successful in a variety of fields, including advanced biological engineering developments^31^, advanced image recognition systems^32^, revolutionary speech recognition technologies^33^, and even modern natural language processing methods. The DNN model is an important tool in contemporary applications because of its adaptability and excellent performance.

The inspiration arises from the excellent outcomes of deep learning models across various fields when tackling complex classification problems. This study investigates the potential of the DNN model in predicting 5hmC sites using a benchmark dataset. Alongside the input and output layers, the proposed DNN model incorporates three hidden layers, as depicted in Fig. 2. Neurons are the fundamental processing units within each model layer, and their numbers vary. Initially, a feature vector $(X {x}_{1}, {x}_{2},{x}_{3},,...{x}_{n})$. The weight vector ${W}_{i}$, and the bias vector Bi is inputted into the input layer, producing the output $Y$ as described in Eq. (12). The first hidden layer receives the output from the input layer, creating a new output. Until the output layer has been reached, the initial hidden layer's output is used as the input for each subsequent hidden layer. Binary data is generated by the output layer, with 1 denoting the existence of 5-hmC samples and 0 denoting their absence.

12 $$Y \, = \, f\left( {XW_{i} + \, B_{i} } \right)$$

The learning model weight vector is built using a He-uniform initialization procedure and is one of its most important parameters. The weight vector is optimized using a backpropagation method and a stochastic gradient. We have used dropped-out and regularization approaches in the suggested model to improve the model's results further and address the problem of model overfitting. Based on its determined values, the activation function is crucial in identifying each neuron's degree of activity. It is an important part of the model. There are several activation functions to select from in the extensive literature. But in this work, the hidden layers used the hyperbolic tangent (Tanh) function, whereas the output layer used the sigmoid function. This purposeful choice was taken to provide a fair and ideal result from the model^34^.

### Activation functions

The activation functions play a critical role in the study and are components for nodes in a neural network; they determine how the output is received and, thus, the model's ability to learn the connected relationships in the data set. The Tanh activation function is utilized in the hidden layers of the study under review, and the output layer is composed of the use of the sigmoid function.

The hyperbolic tangent function, Tanh, is the scale layers' hidden layer selection point because of its symmetrical properties, mapping the input values to a range of [− 1, 1]. This feature is suitable for overcoming the vanishing gradient phenomenon since it offers a way to find both positive and negative relationships, which are essential in big data. The work of study is predefined due to the expressive power of Tanh in the deep internal layers of the Deep Neural Network (DNN). This is so because it enlarges the capability of the model to discover complex patterns and representations. On the output layer, the sigmoid activation function is used, which is part of the model with the nature of binary classification underlying the task at hand. The macro function sigmoid(x) compresses outputs within the range [0, 1], normalizing the results to fit the interpretation of probability for binary outputs. Such selection is especially important when it comes to detecting 5HMC modifications, for instance, where the aim is always a binary classification. Nevertheless, this paper does not deepen the understanding of why these functions were chosen for the particular task, and there needs to be discussion about the alternative functions that could potentially be more efficient for the job. In view of the importance that activation functions play in the performance of the model, more exploration or a clearer articulation of that selection could improve the understanding and clarity of the present study. Debating these options, such as ReLU and Leaky ReLU, together with when and why they should be utilized, would increase the completeness of understanding the implications of the activation function selection in the context of the work.

### Gird search techniques

Grid search is one of the most popular hyperparameter optimization methods in the data science context, and the use of this method to check out the combination of hyperparameters for a given model is called grid search. Within the historical investigation, the DNN model used the grid-search method to improve the parameters of the system. The procedure will first target selecting a number of various points for every hyperparameter in the query, then proceed with recommending a set of combinations that have been exhaustively evaluated in terms of the model’s performance. The end is to pick the best pair of hyperparameter values that will give the most accurate result on a dataset, which is either called 'loss' or 'accuracy' on a validation dataset. The study of naive power or traditional fuel in electricity generation. While the study mentions the use of grid search, it does not provide the specific hyperparameters considered or the range of values explored. Concretely, hyperparameters in a DNN contain learning rate, batch size, number of hidden layers, number of neurons in each layer, activation functions, and regularization parameters. As a way to increase the credibility of the study, the hyperparameter grid search range should be fully mentioned, and the optimized values should then be reported. Such supplementary information will clarify the optimization process details, which other researchers can further use in their extended research with similar parameter search space.

## Criteria for performance evaluation

The following measures have been employed in this study to evaluate the performance of the proposed model: An evaluation of a learning model's performance may be done using a variety of indicators.

*Accuracy* Eq. (13) may be used to calculate the accuracy of a learning model.

13 $$Acc=\frac{{T}^{+}+ {T}^{-}}{{T}^{+} + {F}^{+}+{F}^{-}+{T}^{-}}(100)$$

*Sensitivity* The number of genuine positive classes a classifier recognizes is measured by sensitivity, derived using Eq. (14) and shown as a positive score.

14 $$Sn=\frac{{T}^{+}}{{T}^{+} +{F}^{-}} (100)$$

*Specificity* Using Eq. (15), specificity may be calculated and indicates the negative rate.

15 $$Sp=\frac{{T}^{-}}{{T}^{-} + {F}^{+}} (100)$$

*Mathew's correlation coefficient* The MCC assesses a binary classifier's reliability and corresponds to the:

16 $$Mcc= \frac{({T}^{-}X {T}^{+})-({F}^{+}X {F}^{-})}{\sqrt{({T}^{+} +{F}^{+} )\left({T}^{+}+{F}^{-}\right) ({T}^{-} +{F}^{+} )\left({T}^{-}+{F}^{-}\right) }}$$

*Recall* The F1 score serves as a widely employed tool in the binary classification problem domain to evaluate the model's accuracy. It combines the two at a time and briefly summarizes them, called the F1 score. Eventually, remember that sensitivity (actual positive rate) is a ratio between accurate, optimistic predictions and total real positives. It evaluates the percentage of positive outcomes that a model has recognized as such:

17 $$Recall\frac{{T}^{+}}{{T}^{+}+{F}^{-}}$$

where, ${T}^{+}$ represents true positive 5hmC samples, while ${T}^{-}$, represents true negative 5hmC samples ${F}^{+}$ stands for false positives in 5hmC samples, while ${F}^{-}$, stands for false negatives in 5hmC samples.

## Experimental results and discussion

In this work, we thoroughly evaluated the complete discussion and in-depth analysis of the outcomes produced by the suggested model. Our evaluation considers a wide range of significant elements contributing to its overall success, such as:

1. We meticulously changed the DNN model's setup settings and optimized its performance. This optimization was carried out using an efficient grid search method.
2. We used a variety of feature extraction approaches to objectively assess the model's performance and provide useful insights into its strengths and limitations.
3. We thoroughly evaluated the DNN model's performance compared to popular machine learning models to assess its competitiveness and potential benefits.
4. We meticulously compared the outcomes obtained by Deep5HMC with those of other existing models, enhancing our understanding of its unique contributions and distinguishing characteristics.
5. Finally, we meticulously evaluated the performance of the proposed model by applying it to an independent dataset, ensuring the utmost accuracy and robustness of our evaluation results.

### Optimization of model hyper-parameters

Several parameters need to be configured during the model configuration. These parameters are hyper-parameters and significantly impact the learning model's outcome^35–37^. This study considered the influential parameters presented in Table 2 for optimization. A grid search methodology is adopted to find optimum values of the significant parameters.

**Table 2:** Detailed configuration of proposed DNN Model.

Parameter’s variables	Optimal parameter values
Optimizer	Adam
Dropout	0.4
Regularization L2	0.0001
Number of hidden layers	4
Learning rate	0.01
Seed	1234L
Activation functions	ReLU, Tanh, Sigmoid
Reliable for initializing the weight function	He_Unifrom
Epochs	700
The total number of neurons present in the hidden layers	64, 32, 16, 8

Initially, experiments were carried out to investigate the learning rate's and activation function's impact. Table 3 contains a summary of the experimental results. Analyzing the data in Table 3 reveals that the DNN classifier achieved the highest accuracy of 84.07% when ReLU was used as the activation function and the learning rate was set to 0.01. It is worth noting that lowering the learning rate helps to improve model efficiency; however, reducing it further to 0.01 did not significantly improve the model's performance. As a result, we concluded that ReLU and 0.01, respectively, are the best choices for the activation function and learning rate.

**Table 3:** The DNN model accuracy is evaluated based on activation functions and learning rates.

Learning rate	ReLU	Tanh
0.008	84.05	81.34
0.009	84.02	81.24
**0.01**	**84.07**	**81.44**
0.02	83.08	81.28
0.03	83.38	81.18
0.04	83.09	81.12
0.05	81.19	80.14
0.06	82.70	80.70
0.07	81.76	79.80

Significant values are in bold.

**Figure 3:**
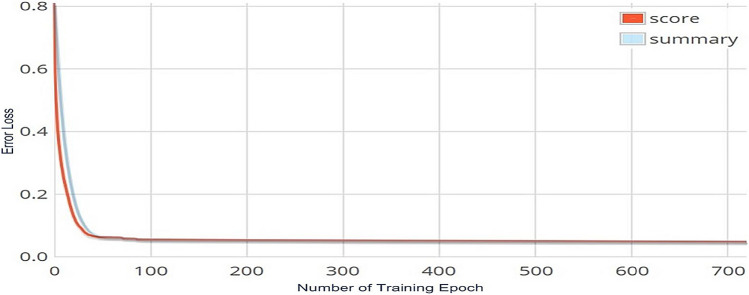
The DNN model error losses across various epochs when using the ReLU activation function.

Secondly, a comprehensive evaluation of the DNN model's performance was carried out by experimenting with different numbers of training epochs. Figure 3 depicts the visual results of this experiment. The figure shows that as the number of training epochs increased, the error losses consistently decreased across all scenarios. The DNN model demonstrated an error loss of 0.8% at the first epoch, which gradually reduced to a remarkable 0.08% as the number of epochs approached 700. However, as the epochs increased, the error losses stabilized, indicating that 700 iterations were the optimal number for achieving the desired result.

### Performance analysis using different sequence formulation methods

This research paper evaluated the proposed DNN model using various feature formulation methods. These methods include Kmer, RC-Kmer, PseDNC, PseTNC, TAC, TCC, and DCC, as displayed in Table 4. The results presented in Table 4 show that the DNN model exhibited superior performance when processing hybrid feature sequences compared to individual feature sequences. For instance, the DNN model achieved an average success rate of 76.87% when utilizing hybrid features, whereas the highest success rate using the specific feature vector (i.e., PseTNC) was 70.94%. The DNN model's improved performance on the hybrid feature vector is its composition of various features, encompassing both local and global correlation information and valuable internal structural information that aids accurate prediction and classification.

**Table 4:** The DNN model performance was completely evaluated using different techniques for sequence formulation.

Methods	Acc%	Sn%	Sp%	F1 score	MCC
Kmer	65.13	71.34	63.23	52.14	0.303
RC-Kmer	67.34	65.43	70.65	53.78	0.294
PseDNC	68.09	67.32	69.33	55.21	0.353
PseTNC	70.94	80.79	69.84	70.03	0.378
TAC	68.95	79.65	67.83	69.08	0.344
TCC	66.32	69.54	73.43	59.92	0.312
DCC	67.87	74.23	63.67	63.80	0.324
Hybrid feature (without feature selection)	76.87	75.75	84.86	76.29	0.584
Hybrid feature (with feature selection)	84.07	90.29	83.07	84.64	0.736

**Figure 4:**
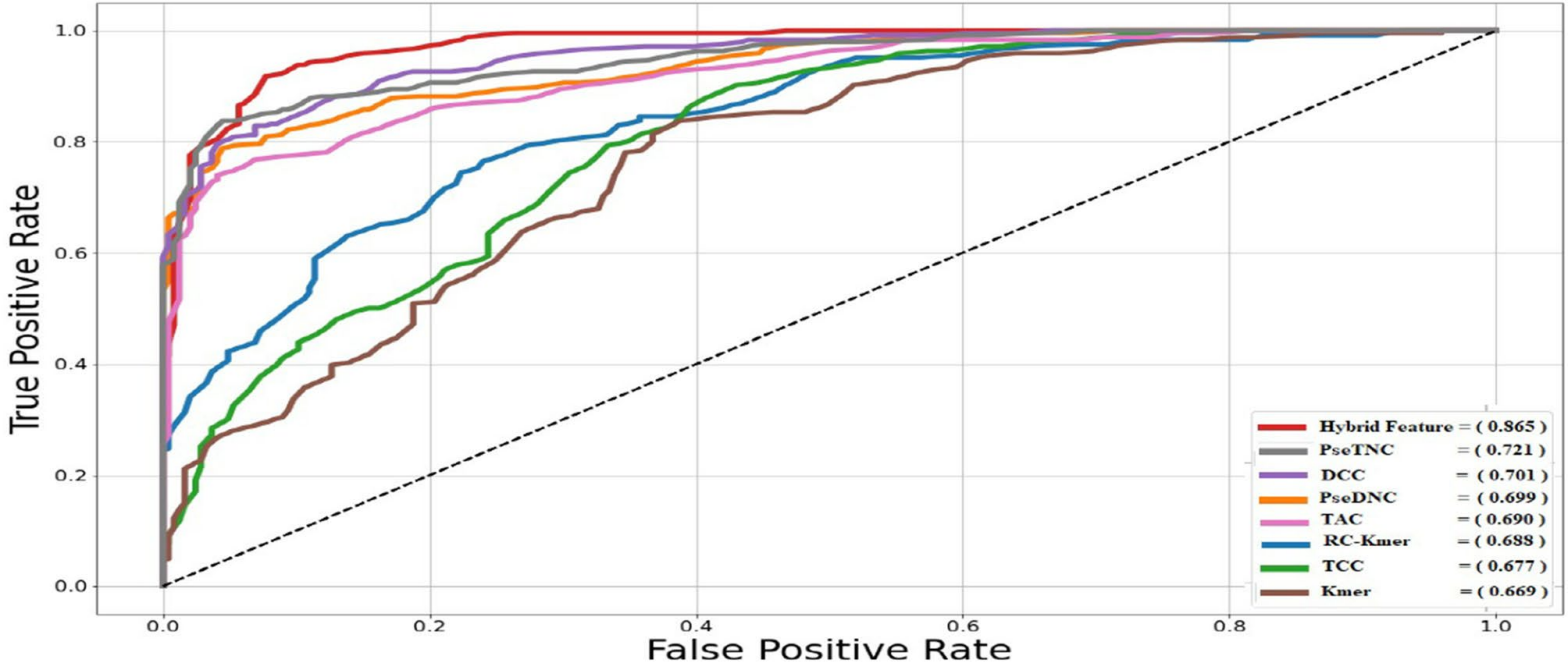
The area under the ROC curve uses different formulation techniques and a hybrid features vector.

In addition, we used a feature selection technique as part of our methodology to reduce the dimensionality of the feature vector. This technique removes unnecessary and noisy features, resulting in a more streamlined and efficient representation. This approach ensures that the selected features are highly informative and contribute significantly to the model's overall performance. This process further improved the DNN efficiency, as shown in Table 4. For example, by using the feature selection method, the model's accuracy significantly improved from 76.87 to 84.07%. Similarly, the other measurement matrices, such as sensitivity (90.29%), specificity (83.07%), and MCC (0.736), also significantly improved, as shown in Table 4. Furthermore, we have adopted the graphical analysis to examine the usefulness of our proposed model, as it is most useful and presented in recent studies of complicated biological systems^37,38^. The value of the receiver operating characteristic (ROC) Curve reflects the model's efficiency so that the higher the value, the better the output. Figure 4 shows the graph of the ROC curve (AUC). As shown in Fig. 4, the proposed classifier using a hybrid feature (with feature selection) has a remarkably larger ROC value, i.e., 0.865, compared with the single feature extraction method. This statistic indicates that the proposed model has more than 86% capability to discriminate between positive and negative classes accurately.

### Performance comparison of different classifiers

In this paper, we examined the proposed predictor's effectiveness in-depth by comparing its performance to that of widely used classifiers^38,39^. A hybrid features vector was used in the evaluation. It used well-known classifiers like KNN^40,41^, Random Forest (RF)^42^, Naive Bayes (NB)^43^, and Support-Vector-Machine (SVM)^44^. Table 5 displays the results of this comparative analysis, highlighting the performance of each learning classifier. Additionally, our proposed DNN model outperformed all other classifiers, with an average accuracy of 84.07%. Among the classifiers mentioned, KNN had the second-highest accuracy at 79.89%, while the RF classifier had the lowest accuracy at 62.30%. A careful examination of these results led us to conclude that our proposed model's exceptional performance can be attributed to its ability to process complex and highly nonlinear datasets effectively using its multi-stack processing capabilities.

**Table 5:** A comparative analysis assessed the proposed model performance compared to various learning classifiers.

Learning classifiers	NB	SVM	KNN	RF	DT	DNN
ACC%	72.24	63.94	79.89	62.3	77.08	84.07
SN%	78.8	61.24	72.82	61.51	74.34	90.29
SP%	68.4	63.73	85.15	58.08	84.5	83.07
F1 score	70.61	57.81	76.65	56.95	73.33	84.64
MCC	0.377	0.296	0.443	0.317	0.395	0.736

### Performance comparison with existing models

In this study, we have compared the performance of the suggested model with the existing methods, i.e.,^45–49^. Table 6 provides a comparison between the proposed model and the current models. It can be observed from Table 6 that the suggested model performed overwhelmingly better than the current model. The suggested predictor achieved the highest accuracy of 84.07% and Matthews correlation coefficient (MCC) of 0.736, respectively. These important metrics reflect the proposed predictor's overall performance, robustness, and stability. The suggested model also yields much better performances in Sn (sensitivity) and Sp (specificity) comparable with the existing methods, i.e., 90.29% and 83.07%, respectively. The average accuracy improvements, i.e., 7.59%, illustrate the proposed model's significance and self-evident comparison with existing predictors.

**Table 6:** The proposed and existing models were compared concerning performance.

Methods	iRNA5hmC	iRNA5hmC-PS	iRhm5CNN	Deep5hmC
Classifier	SVM	LR	CNN	DNN
ACC (%)	65.48	78.3	81	84.07
SN (%)	67.67	80	82	90.29
SP (%)	63.29	79.5	80	83.07
F1 score	65.01	76.41	78.21	83.94
MCC	0.31	0.56	0.62	0.736

### Analysis of learning hypotheses using independent dataset

We extensively evaluated using an independent dataset to ensure our groundbreaking model's highest stability and unwavering reliability. The highly detailed and meticulously organized results in Table 7 provide a profound understanding of the hybrid feature set's remarkable performance across diverse classifiers. It is especially noteworthy that our pioneering DNN classifier demonstrated unparalleled accuracy, achieving an astounding 83.31%. Furthermore, it outperformed all other classifiers by performing an amazing 90.01% sensitivity and an exceptional 82.98% specificity. In addition, on the composite feature set, the KNN classification served well, attaining the second-highest accuracy and special MCC values of 78.49 and 0.438.

**Table 7:** The proposed model's performance on an independent dataset was evaluated and analyzed.

Classifiers	ACC (%)	SN (%)	SP (%)	F1 score	MCC
NB	70.32	77.22	67.55	73.26	0.372
SVM	61.41	60.11	62.31	59.25	0.293
DT	75.98	73.23	83.15	76.47	0.394
RF	60.21	61.01	56.89	60.92	0.316
KNN	78.49	73.27	80.21	77.73	0.438
DNN	83.31	90.01	83.98	85.57	0.732

## Conclusion

Several biological processes, such as gene regulation and epigenetic changes, necessitate the identification of 5-hydroxymethylcytosine (5hmC) modifications. These alterations have been associated with conditions like diabetes, cardiovascular disease, and cancer, highlighting the importance of precisely defining 5HMC regions for early disease detection and diagnosis. The proposed Deep5HMC model offers a more efficient and cost-effective alternative to traditional laboratory techniques for locating 5HMC samples, employing machine learning algorithms and discriminative feature extraction methodologies. This study introduces Deep5HMC as an effective and efficient learning model for identifying 5HMC samples, leveraging machine learning approaches, discriminative feature extraction methods, and deep neural networks to enhance prediction precision and robustness. The trials revealed a notable improvement in Deep5HMC's prediction accuracy, reaching 84.07%, surpassing previous investigations' recognition accuracy of 7.59%. These findings demonstrate the superior performance of the optimized DNN classification method. Exploring potential combinations of Deep5HMC with other computational or experimental techniques holds promise for enhancing RNA modification analysis. Future work directions aim to enhance Deep5HMC's continuous improvement and practicality in RNA modification analysis using complementary computational methods.

## Data Availability

The datasets used and/or analyzed during the current study are available on Github link. https://github.com/salman-khan-mrd/Deep5HMC.

## References

[1] Brosius, J. & Raabe, C. A. What is an RNA? A top layer for RNA classification. *RNA Biol.* **13**, 140–144. 10.1080/15476286.2015.1128064 (2016). 26818079 10.1080/15476286.2015.1128064 PMC4829331

[2] Thiel, V., Herold, J., Schelle, B. & Siddell, S. G. Infectious RNA transcribed in vitro from a CDNA copy of the human coronavirus genome cloned in vaccinia virus. *J. Gen. Virol.* **82**, 1273–1281. 10.1099/0022-1317-82-6-1273 (2001). 11369870 10.1099/0022-1317-82-6-1273

[3] Williams, G. D., Gokhale, N. S. & Horner, S. M. Regulation of viral infection by the RNA modification N6-methyladenosine. *Annu. Rev. Virol.* **6**, 235–253. 10.1146/annurev-virology-092818-015559 (2019). 31283446 10.1146/annurev-virology-092818-015559 PMC6884077

[4] Uemura, Y., Hasegawa, A., Kobayashi, S. & Yokomori, T. Tree adjoining grammars for RNA structure prediction. *Theor. Comput. Sci.* **210**, 277–303. 10.1016/S0304-3975(98)00090-5 (1999).

[5] Chen, W., Feng, P., Song, X., Lv, H. & Lin, H. IRNA-M7G: Identifying N7-methylguanosine sites by fusing multiple features. *Mol. Ther. Nucleic Acids* **18**, 269–274. 10.1016/j.omtn.2019.08.022 (2019). 31581051 10.1016/j.omtn.2019.08.022 PMC6796804

[6] Conde, J., Yoon, J.-H., Roy Choudhury, J., Prakash, L. & Prakash, S. Genetic control of replication through N1-methyladenine in human cells. *J. Biol. Chem.* **290**, 29794–29800. 10.1074/jbc.M115.693010 (2015). 26491020 10.1074/jbc.M115.693010 PMC4705973

[7] Liu, Z. Y. *et al.* Le MDR: An integrative DNA N6-methyladenine and N4-methylcytosine modification database for rosaceae. *Hortic. Res.* **6**, 1–6. 10.1038/s41438-019-0160-4 (2019). 31240103 10.1038/s41438-019-0160-4 PMC6572862

[8] Fu, L. *et al.* Tet-mediated formation of 5-hydroxymethylcytosine in RNA. *J. Am. Chem. Soc.* **136**, 11582–11585. 10.1021/ja505305z (2014). 25073028 10.1021/ja505305z PMC4140497

[9] Huber, S. M. *et al.* Formation and abundance of 5-hydroxymethylcytosine in RNA. *ChemBioChem* **16**, 752–755. 10.1002/cbic.201500013 (2015). 25676849 10.1002/cbic.201500013 PMC4471624

[10] Roundtree, I. A., Evans, M. E., Pan, T. & He, C. Dynamic RNA modifications in gene expression regulation. *Cell* **169**, 1187–1200. 10.1016/j.cell.2017.05.045 (2017). 28622506 10.1016/j.cell.2017.05.045 PMC5657247

[11] Uribe-Lewis, S. *et al.* 5-Hydroxymethylcytosine and gene activity in mouse intestinal differentiation. *Sci. Rep.* **10**, 546. 10.1038/s41598-019-57214-z (2020). 31953501 10.1038/s41598-019-57214-z PMC6969059

[12] Ahmad, A. *et al.* Deep-AntiFP: Prediction of antifungal peptides using distanct multi-informative features incorporating with deep neural networks. *Chemom. Intell. Lab. Syst.* **208**, 104214. 10.1016/j.chemolab.2020.104214 (2021).

[13] Dong, Z. W. *et al.* RTL-P: A sensitive approach for detecting sites of 2′-O-methylation in RNA molecules. *Nucleic Acids Res.*10.1093/nar/gks698 (2012). 22833606 10.1093/nar/gks698 PMC3488209

[14] Inayat, N. *et al.* IEnhancer-DHF: Identification of enhancers and their strengths using optimize deep neural network with multiple features extraction methods. *IEEE Access* **9**, 40783–40796. 10.1109/ACCESS.2021.3062291 (2021).

[15] Ali, S. D., Kim, J. H., Tayara, H. & Chong, K. T. Prediction of RNA 5-hydroxymethylcytosine modifications using deep learning. *IEEE Access* **9**, 8491–8496. 10.1109/ACCESS.2021.3049146 (2021).

[16] Khan, S. *et al.* Optimized feature learning for anti-inflammatory peptide prediction using parallel distributed computing. *Appl. Sci.* **13**, 7059. 10.3390/app13127059 (2023).

[17] Majid, A., Khan, M. M., Iqbal, N., Jan, M. A. & Khan, M. M. Salman application of parallel vector space model for large-scale DNA sequence analysis. *J. Grid Comput.* **17**, 313–324. 10.1007/s10723-018-9451-5 (2019).

[18] Liu, Y., Chen, D., Su, R., Chen, W. & Wei, L. IRNA5hmC: The first predictor to identify RNA 5-hydroxymethylcytosine modifications using machine learning. *Front. Bioeng. Biotechnol.* **8**, 1–8. 10.3389/fbioe.2020.00227 (2020). 32296686 10.3389/fbioe.2020.00227 PMC7137033

[19] Chen, Z. *et al.* ILearn: An integrated platform and meta-learner for feature engineering, machine-learning analysis and modeling of DNA, RNA and protein sequence data. *Brief. Bioinform.* **21**, 1047–1057. 10.1093/bib/bbz041 (2020). 31067315 10.1093/bib/bbz041

[20] Lin, H., Deng, E. Z., Ding, H., Chen, W. & Chou, K. C. IPro54-PseKNC: A sequence-based predictor for identifying sigma-54 promoters in prokaryote with pseudo k-tuple nucleotide composition. *Nucleic Acids Res.* **42**, 12961–12972. 10.1093/nar/gku1019 (2014). 25361964 10.1093/nar/gku1019 PMC4245931

[21] Liu, Z., Xiao, X., Qiu, W.-R.R. & Chou, K.-C.C. IDNA-methyl: Identifying DNA methylation sites via pseudo trinucleotide composition. *Anal. Biochem.* **474**, 69–77. 10.1016/j.ab.2014.12.009 (2015). 25596338 10.1016/j.ab.2014.12.009

[22] Khan, S. *et al.* A two-level computation model based on deep learning algorithm for identification of PiRNA and their functions via Chou’s 5-steps rule. *Int. J. Pept. Res. Ther.* **26**, 795–809. 10.1007/s10989-019-09887-3 (2020).

[23] Chen, Z. *et al.* ILearnPlus: A comprehensive and automated machine-learning platform for nucleic acid and protein sequence analysis prediction and visualization. *Nucleic Acids Res.*10.1093/nar/gkab122 (2021). 33660783 10.1093/nar/gkab122 PMC8191785

[24] Khan, S., Naeem, M. & Qiyas, M. Deep intelligent predictive model for the identification of diabetes. *AIMS Math.* **8**, 16446–16462. 10.3934/math.2023840 (2023).

[25] Khan, S., Khan, M., Iqbal, N., Amiruddin Abd Rahman, M. & Khalis Abdul Karim, M. Deep-PiRNA: Bi-layered prediction model for PIWI-interacting RNA using discriminative features. *Comput. Mater. Contin.* **72**, 2243–2258. 10.32604/cmc.2022.022901 (2022).

[26] Ben-Bassat, I., Chor, B. & Orenstein, Y. A deep neural network approach for learning intrinsic protein-RNA binding preferences. *Bioinformatics* **34**, i638–i646. 10.1093/bioinformatics/bty600 (2018). 30423078 10.1093/bioinformatics/bty600

[27] Khan, F. *et al.* Prediction of recombination spots using novel hybrid feature extraction method via deep learning approach. *Front. Genet.* **11**, 1052. 10.3389/fgene.2020.539227 (2020). 10.3389/fgene.2020.539227 PMC7527634 33093842

[28] Khan, S., Khan, M., Iqbal, N., Khan, S. A. & Chou, K.-C. Prediction of PiRNAs and their function based on discriminative intelligent model using hybrid features into Chou’s PseKNC. *Chemom. Intell. Lab. Syst.* **203**, 104056. 10.1016/j.chemolab.2020.104056 (2020).

[29] Ravi, D. *et al.* Deep learning for health informatics. *IEEE J. Biomed. Heal. Inform.* **21**, 4–21. 10.1109/JBHI.2016.2636665 (2017). 10.1109/JBHI.2016.2636665 28055930

[30] Ma, J., Sheridan, R. P., Liaw, A., Dahl, G. E. & Svetnik, V. Deep neural nets as a method for quantitative structure–activity relationships. *J. Chem. Inf. Model.* **55**, 263–274. 10.1021/ci500747n (2015). 25635324 10.1021/ci500747n

[31] Zhu, Z. *et al.* Deep learning for identifying radiogenomic associations in breast cancer. *Comput. Biol. Med.* **109**, 85–90. 10.1016/j.compbiomed.2019.04.018 (2019). 31048129 10.1016/j.compbiomed.2019.04.018 PMC7155381

[32] Krizhevsky, A., Sutskever, I. & Hinton, G. E. ImageNet classification with deep convolutional neural networks. *Commun. ACM* **60**, 84–90. 10.1145/3065386 (2017).

[33] Hinton, G. *et al.* Deep neural networks for acoustic modeling in speech recognition: The shared views of four research groups. *IEEE Signal Process. Mag.* **29**, 82–97. 10.1109/MSP.2012.2205597 (2012).

[34] Wang, B. *et al.* Deep neural nets with interpolating function as output activation. *Proc. Adv. Neural Inf. Process. Syst.* **2018**, 743–753 (2018).

[35] Liu, B., Yang, F. & Chou, K. C. 2L-PiRNA: A two-layer ensemble classifier for identifying piwi-interacting RNAs and their function. *Mol. Ther. Nucleic Acids* **7**, 267–277. 10.1016/j.omtn.2017.04.008 (2017). 28624202 10.1016/j.omtn.2017.04.008 PMC5415553

[36] Liu, B., Wu, H. & Chou, K.-C. Pse-in-one 2.0: An improved package of web servers for generating various modes of pseudo components of DNA, RNA, and protein sequences. *Nat. Sci.* **09**, 67–91. 10.4236/ns.2017.94007 (2017).

[37] Bao, W., Gu, Y., Chen, B. & Yu, H. Golgi_DF: Golgi proteins classification with deep forest. *Front. Neurosci.* **12**(17), 1197824 (2023). 10.3389/fnins.2023.1197824 PMC10213405 37250391

[38] Bao, W., Cui, Q., Chen, B. & Yang, B. Phage_UniR_LGBM: Phage virion proteins classification with UniRep features and LightGBM model. *Comput. Math. Methods Med.* **15**, 2022 (2022). 10.1155/2022/9470683 PMC9033350 35465015

[39] Bao, W., Liu, Y. & Chen, B. Oral_voting_transfer: classification of oral microorganisms’ voting transfer model. *Front. Microbiol.* **7**(14), 1277121 (2024). 10.3389/fmicb.2023.1277121 PMC10879614 38384719

[40] Zhou, G. P. & Deng, M. H. An extension of Chou’s graphic rules for deriving enzyme kinetic equations to systems involving parallel reaction pathways. *Biochem. J.* **222**, 169–176. 10.1042/bj2220169 (1984). 6477507 10.1042/bj2220169 PMC1144157

[41] Le, N. Q., Do, D. T. & Le, Q. A. A sequence-based prediction of Kruppel-like factors proteins using XGBoost and optimized features. *Gene.* **30**(787), 145643 (2021). 10.1016/j.gene.2021.145643 33848577

[42] Yuan, Q., Chen, K., Yu, Y., Le, N. Q. & Chua, M. C. Prediction of anticancer peptides based on an ensemble model of deep learning and machine learning using ordinal positional encoding. *Brief. Bioinform.* **24**(1), 630 (2023). 10.1093/bib/bbac630 36642410

[43] Chou, K. C. & Forsén, S. Graphical rules for enzyme-catalysed rate laws. *Biochem. J.* **187**, 829–835. 10.1042/bj1870829 (1980). 7188428 10.1042/bj1870829 PMC1162468

[44] Cheng, D., Zhang, S., Deng, Z., Zhu, Y. & Zong, M. KNN algorithm with data-driven k value. In *Proceedings of the Advanced Data Mining and Applications* (eds Luo, X. *et al.*) 499–512 (Springer, 2014).

[45] Fawagreh, K., Gaber, M. M. & Elyan, E. Random forests: From early developments to recent advancements. *Syst. Sci. Control Eng.* **2**, 602–609. 10.1080/21642583.2014.956265 (2014).

[46] Myaeng, S. H., Han, K. S. & Rim, H. C. Some effective techniques for Naive Bayes text classification. *IEEE Trans. Knowl. Data Eng.* **18**, 1457–1466. 10.1109/TKDE.2006.180 (2006).

[47] Yue, S., Li, P. & Hao, P. SVM classification: Its contents and challenges. *Appl. Math. J. Chinese Univ.* **18**, 332–342. 10.1007/s11766-003-0059-5 (2003).

[48] Ahmed, S. *et al.* Accurate prediction of RNA 5-hydroxymethylcytosine modification by utilizing novel position-specific gapped k-Mer descriptors. *Comput. Struct. Biotechnol. J.* **18**, 3528–3538. 10.1016/j.csbj.2020.10.032 (2020). 33304452 10.1016/j.csbj.2020.10.032 PMC7701324

[49] Khan, S. *et al.* Enhancing sumoylation site prediction: A deep neural network with discriminative features. *Life.* **13**, 2153. 10.3390/life13112153 (2023). 38004293 10.3390/life13112153 PMC10672286

